# From expert opinion to data driven selection of sports equipment: Boot selection in alpine ski racers

**DOI:** 10.1371/journal.pone.0349862

**Published:** 2026-06-04

**Authors:** Christina Kranzinger, Thomas Stöggl, Helmut Holzer, Josef Kröll, Michael Lasshofer

**Affiliations:** 1 Salzburg Research Forschungsgesellschaft mbH, Salzburg, Austria; 2 Department of Sport and Exercise Science, University of Salzburg, Salzburg, Austria; 3 Red Bull Athlete Performance Center Salzburg, Salzburg, Austria; 4 Atomic Austria GmbH, Altenmarkt, Austria; World Health Organization, Regional Office for South-East Asia, INDIA

## Abstract

To compete successfully in alpine ski racing, not only physical, mental, and skiing technique preparation are important, but also wisely chosen and properly tuned skiing equipment. While equipment related safety aspects are mostly ensured by official rules (e.g., competition rules provided by the International Skiing Federation), the performance optimization and customization of equipment is mostly reliant on coaches and experts in the skiing industry. Therefore, knowledge often is dependent on individual person’s experience and not publicly available. This aspect can also hinder companies or teams to spread knowledge within their own team. However, there is the necessity to select the optimal equipment for each athlete individually within a range of available equipment variations. To improve the process of ski boot selection for racers within a whole company, the presented research had the goal to provide a decision model by extracting and objectifying knowledge from few experts within the company. Based on a Delphi-type expert elicitation approach, together with the company’s boot experts a data collection sheet was designed, including the relevant parameter to decide which ski boot is appropriate for an individual athlete. Thereafter, data analysis of 198 datasets included classification methods based on random forest models to create boot choice recommendation models. Results revealed well predictable recommendation for boot size (accuracy 77%) and acceptable accuracy (57%) for prediction of boot model. With existing limitations of extracting subjective and individual expertise, this approach helps to objectify personalized expertise and distribute knowledge within relevant interest groups.

## Introduction

Alpine ski racing is a multifaceted winter sport with a wide variety of influence on sporting performance and injury risk [1], with both being inseparable in many cases. As a key component, sport equipment (e.g., system of boot, binding, plate, and ski) was named most frequently as a key injury risk factor [2,3]. As a result, well-chosen and adjusted sport equipment is crucial. Within other sports, like running [4,5], cycling [6,7] or ice speed skating [8,9] the importance of equipment adaptation and individualization has already been demonstrated. It was also shown that young athletes can be supported in learning new motor skills safely with adapted, scaled and well fitted equipment and playgrounds irrespective of the sport [10].

The International Ski Federation (FIS) and the national skiing federations provide mandatory equipment requirements not only for elite World Cup skiers, but also scaled equipment regulations for youth and kids racing series. While these regulations build the framework, ski racing suppliers provide not only equipment serving these regulations in different disciplines (slalom, giant slalom, super-g, downhill), but also serving individual preferences by providing different options regarding for example stiffness, fit, comfort, or skis’ bending characteristics. This results in the availability of a wide range of skis, plates, bindings and boots.

While the regulation of skis (e.g., length, width, and sidecut radius) changes throughout age groups, the regulation of boots is mostly limited to standing height and does not change. Accordingly, a wide range of boots differing in nominal flex index (NFI) as a marker of stiffness, plastic composition, and various geometrical aspects is available. In the present study, this variety was represented by ten boot models and eight boot sizes.

Linked to the non-standardized parameter NFI, general recommendations suggest to choose stiffer boots (higher NFI) with increasing skiing level and for heavier athletes [11]. Additionally, the selection of boot size is not only relevant in terms of boot length, but also in terms of the width since different boot models have different width measures.

With respect to an appropriate boot choice, these before mentioned available boot features should then be set into context of individual athlete’s characteristics, measures, and skiing style related needs. Given this variety, the “In-store” choice of boot cannot solely be based on a metric like the NFI and foot length measurement but needs a high level of experience as well (i.e., from the boot technician). This process then is conventionally accompanied by extensive and time-consuming comparative on-snow testing, to verify whether the equipment/boot choice was adequate. This technician centered approach can make athletes dependent on single experts with little chance to compare to an objective and overarching standard or other experts. To optimize this process, and given the undisputed importance of boot choice for safety and performance [3,12,13], this study aimed to define and validate key parameters together with experts. The goal was to base ski racing boot selection on measurable variables rather than solely on subjective experience, which, to the best of our knowledge, has not been done before. The hypothesis was, that experts’ knowledge and experience can be explored and extracted by defining and collecting objective parameters combined with skiing style related items and compute them with a modelling approach to predict the boot choice for alpine ski racers with acceptable accuracy.

Despite the central role of ski boot selection in performance and safety, no systematic or empirically derived framework exists to guide this process. Current practice relies heavily on the subjective judgment of individual boot technicians. In alpine skiing, expert fitting procedures are not standardized, expert knowledge is rarely documented, and decision criteria remain largely implicit rather than measurable or reproducible. This lack of transparency limits internal consistency, complicates knowledge transfer within companies, and makes objective evaluation of decision quality impossible. The aim of this study was therefore to translate the implicit decision criteria of experienced boot technicians into measurable variables and to model them in a way that allows for consistent and reproducible recommendations for boot size and boot model in form of a proof-of-concept for knowledge extraction and formalization within a specific brand. Addressing this question fills a critical gap by transforming tacit expertise into an explicit, data-driven decision structure, providing a foundation for more standardized and transparent equipment selection.

## Materials and methods

### Development of the datasheet

The datasheet was developed together with the ski boot experts from the Atomic Pro Center in Austria, to cover all important information to choose a ski boot.

The generation of the datasheet was based on a structured expert consultation inspired by Delphi-type expert elicitation approaches [14] and consisted of three main stages. In stage one, four Atomic boot experts with several years of experience in providing equipment for alpine ski racers were interviewed individually. All items they identified as potentially influencing boot choice were recorded.

During stage two, guided by a researcher with additional experience and qualifications as a ski racing coach, all items and the associated argument of relevance were discussed within the group of experts. Stage three included the creation of a consensus datasheet, implementing all items the experts agreed on. Grouped categories included anthropometric data (biological sex, age, body height, body weight, foot length, foot width, instep height), characteristics of the old ski boot (model, NFI, size), skiing related items (skiing discipline and skiing style related items: vertical movement of the athlete, body position in sagittal plane and chosen line between the gates) and a boot recommendation based on available boot models and boot sizes. This datasheet can be found in the Supporting information.

### Data collection

As a next step, the boot experts from the datasheet generation process plus one colleague (n = 5) were asked to use the datasheet whenever they equipped a ski racer in the in-house performance center. The customers of this performance center are racers of any competition category, including kids, junior racers as well as adult racers and masters racers. Competition categories were taken from the Austrian Competition Rules (Kids = 7–12 years of age; Juniors 13–16 years of age; FIS > 16 years of age). In addition, no professional ski racers are equipped in this center, as they are provided highly individualized and often prototype-state equipment. Data collection took place over two consecutive years (1.7.2020 to 30.11.2021). All participants provided written informed consent and the study was granted by the Ethics Committee of the Salzburg University (EK-GZ: 11/2018).

### Prediction model

For each of the two recommendations (boot size and boot model) a separate predictive model was developed. As classification method, random forest models were chosen [15], since this method enables to identify most important variables and detailed class distribution. This provides the possibility to recommend not only one boot size or boot model, but also the detailed class distribution and the probabilities for the second most likely choice. For development of the model, the questionnaire data of 2020 and 2021 were combined to increase sample size and robustness of the model. To include records with missing values, the missing values were imputed via Multivariate Imputation by Chained Equations (mice) [16]. The three skiing-style variables were originally assessed on a five-point Likert scale but were collapsed into three categories for analysis by combining levels 1–2 and 4–5.

For example, the race line variable originally included five levels (“very round”, “rather round”, “neutral”, “rather direct”, “very direct”) which were grouped into three categories (“round”, “neutral”, “direct”).

This decision was made for two reasons. First, preliminary inspection of the data showed that some of the five categories contained only very few observations, resulting in sparse and unstable factor levels. Collapsing adjacent categories (e.g., “very round” + “rather round”) improved the balance between levels and prevented the model from being influenced by extremely small subgroups. Second, expert feedback indicated that, in practical boot selection, technicians do not distinguish meaningfully between all five levels of these descriptors; instead, they interpret movement patterns in broader qualitative categories (e.g., “round” / “neutral” / “direct line”).

Categories with extremely low sample sizes (n smaller than 10) (e.g., “masters”) were removed prior to model training, as such sparse classes do not allow reliable learning and may introduce instability in classification models due to class imbalance.

**Fig 1:**
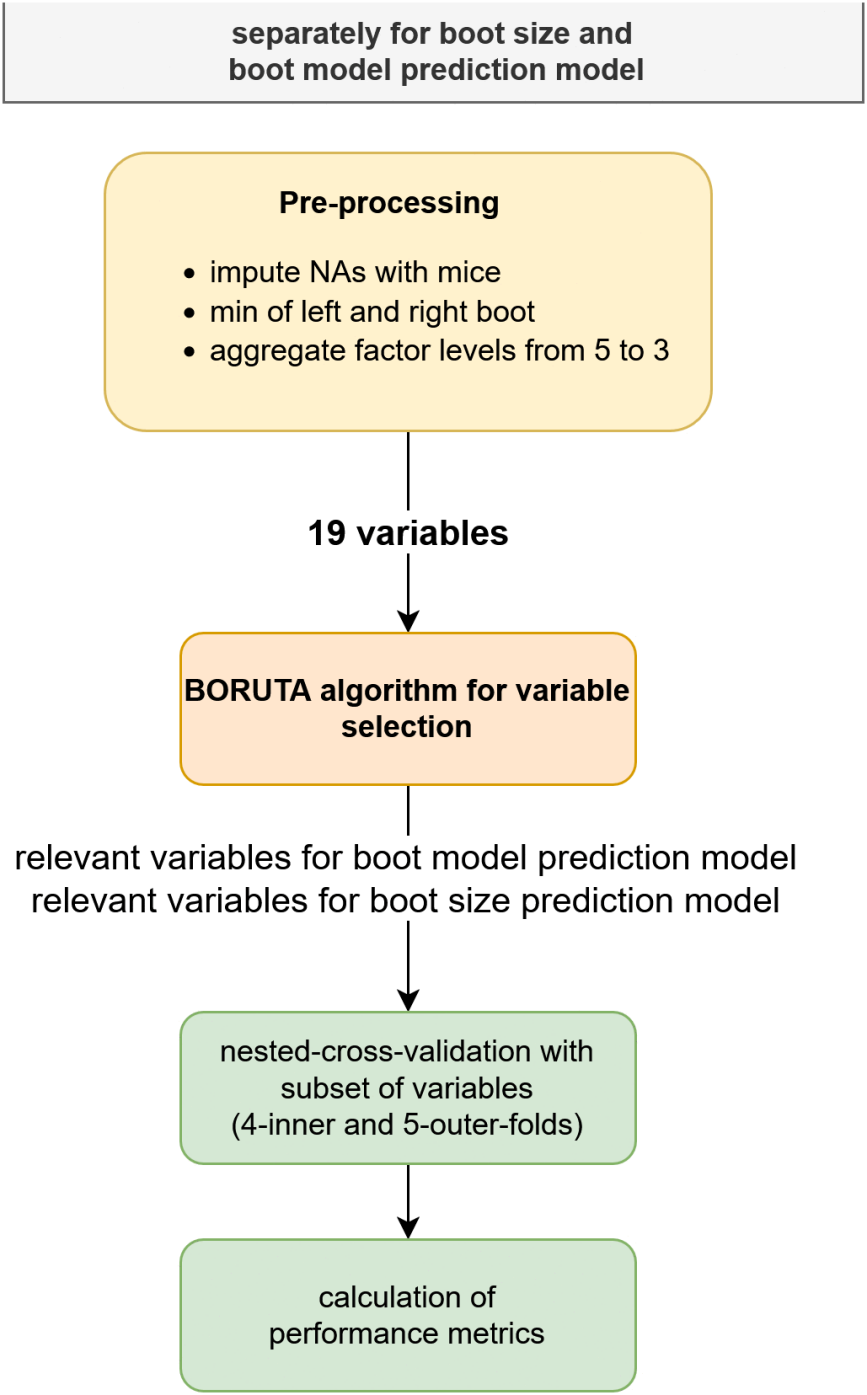
Data analysis process. Overview of the data analysis process.

For bilaterally measured variables like foot length, foot width or instep height, the minimum value between left and right was used. Boot experts stated concordantly that they can lengthen the boot for at least one size with special tuning tools, but it is not possible to make a boot smaller. Same principle also applies to boot width. Therefore, in case of side differences, the smaller values were considered for analysis. In total, 19 variables were available for data analysis. The Boruta algorithm [17] was applied for variable selection, which identifies the most important variables used in the final random forest model. The Boruta algorithm is also based on random forest models, but adds shuffled copies of all variables, so-called shadows, to create random fluctuation in an extended data set. The shadow variables were subsequently used to decide which variables are important [17]. A random forest is a popular algorithm that is based on multiple independent trees. Each tree is created randomly by using a bootstrap sample of the training set and at each node, the best split among a random subset of features is used to split the node into two daughter nodes [18]. Fig 1 gives an overview of the analytical process. To tune the hyperparameters and estimate the random forests’ performances a nested-cross-validation with four inner- and five outer-folds was used. Hyperparameter tuning was performed using the *caret* package [18] within the inner cross-validation loop. Specifically, the mtry parameter was optimized using the default tuning procedure of *caret*, which generates a grid of candidate values based on the number of predictors, while the number of trees was kept at its default value (ntree = 500).

Mean accuracy, mean No Information Rate (NIR) and mean Cohen’s Kappa [19] values are reported and interpreted to evaluate the performance of the prediction models. For the boot size models weighted Cohen’s Kappa, for the boot recommendation model unweighted Cohen’s Kappa was used. Compared to simple accuracy Cohen’s Kappa is a performance measure where random accuracy is removed. Cohen’s Kappa ranges from −1, which can be interpreted as a result worse than obtained randomly, to +1, which can be interpreted as perfect classification [20]. NIR corresponds to the proportion of the most frequent class in the dataset and reflects the accuracy obtained by always predicting this majority class.

The models were calculated for all skiers together and additionally for female and male skiers separately. All models were computed with R [21] and the packages caret [18], mice [16] and random forest [22]. The package Boruta [17] was used for variable selection.

## Results

### Overall results

A total of 198 participants (40% females, 60% males), 45 were kids, 98 juniors and 55 FIS level racers, were evaluated. Sample size, anthropometric data for each age group and biological sex is presented in Table 1.

**Table 1:** Sample size, age and anthropometric data (mean ± SD) for each age-group.

Group	Variable	Overall	Female	Male
Kids	Sample size	45	16	29
	Age [years]	10.5 ± 0.9	10.4 ± 0.7	10.6 ± 0.9
	Body weight [kg]	38.2 ± 6.1	37.8 ± 4.9	38.4 ± 6.6
	Body height [m]	1.47 ± 0.07	1.47 ± 0.06	1.48 ± 0.06
Juniors	Sample size	98	36	62
	Age [years]	13.6 ± 1.2	13.8 ± 1.3	13.5 ± 1.2
	Body weight [kg]	51.9 ± 9.7	51.6 ± 8.3	52.1 ± 10.5
	Body height [m]	1.63 ± 0.09	1.61 ± 0.09	1.64 ± 0.10
FIS	Sample size	55	26	29
	Age [years]	17.2 ± 1.3	17.2 ± 1.2	17.3 ± 1.4
	Body weight [kg]	66.8 ± 9.3	60.5 ± 4.3	72.6 ± 8.8
	Body height [m]	1.73 ± 0.07	1.68 ± 0.06	1.78 ± 0.05

Table 2 displays the performance metrics for all random forest models, the model calculated with all skiers and the models calculated for female and male skiers separately. Results also include range of accuracy and range of Cohen’s Kappa. The hyperparameters used for the random forest models are listed in detail in the Supporting information.

**Table 2:** Performance metrics for all recommendation prediction models (mean over all 5 folds ± SD, Acc: Accuracy, NIR: No Information Rate, CK: (weighted) Cohen’s Kappa).

	Overall		Female		Male	
Metric	Size	Model	Size	Model	Size	Model
Acc.	0.77 ± 0.05	0.57 ± 0.06	0.75 ± 0.10	0.64 ± 0.08	0.68 ± 0.06	0.57 ± 0.17
Acc. range	0.72–0.83	0.51–0.64	0.60–0.87	0.56–0.75	0.58–0.75	0.38–0.79
NIR	0.25 ± 0.01	0.21 ± 0.01	0.36 ± 0.03	0.37 ± 0.02	0.25 ± 0.00	0.22 ± 0.03
NIR range	0.23–0.26	0.19–0.23	0.33–0.40	0.33–0.40	0.25–0.25	0.21–0.25
CK.	0.86 ± 0.07	0.50 ± 0.07	0.75 ± 0.11	0.52 ± 0.08	0.81 ± 0.06	0.51 ± 0.19
CK. range	0.83–0.89	0.43–0.59	0.56–0.87	0.43–0.75	0.72–0.84	0.29–0.79

**Fig 2:**
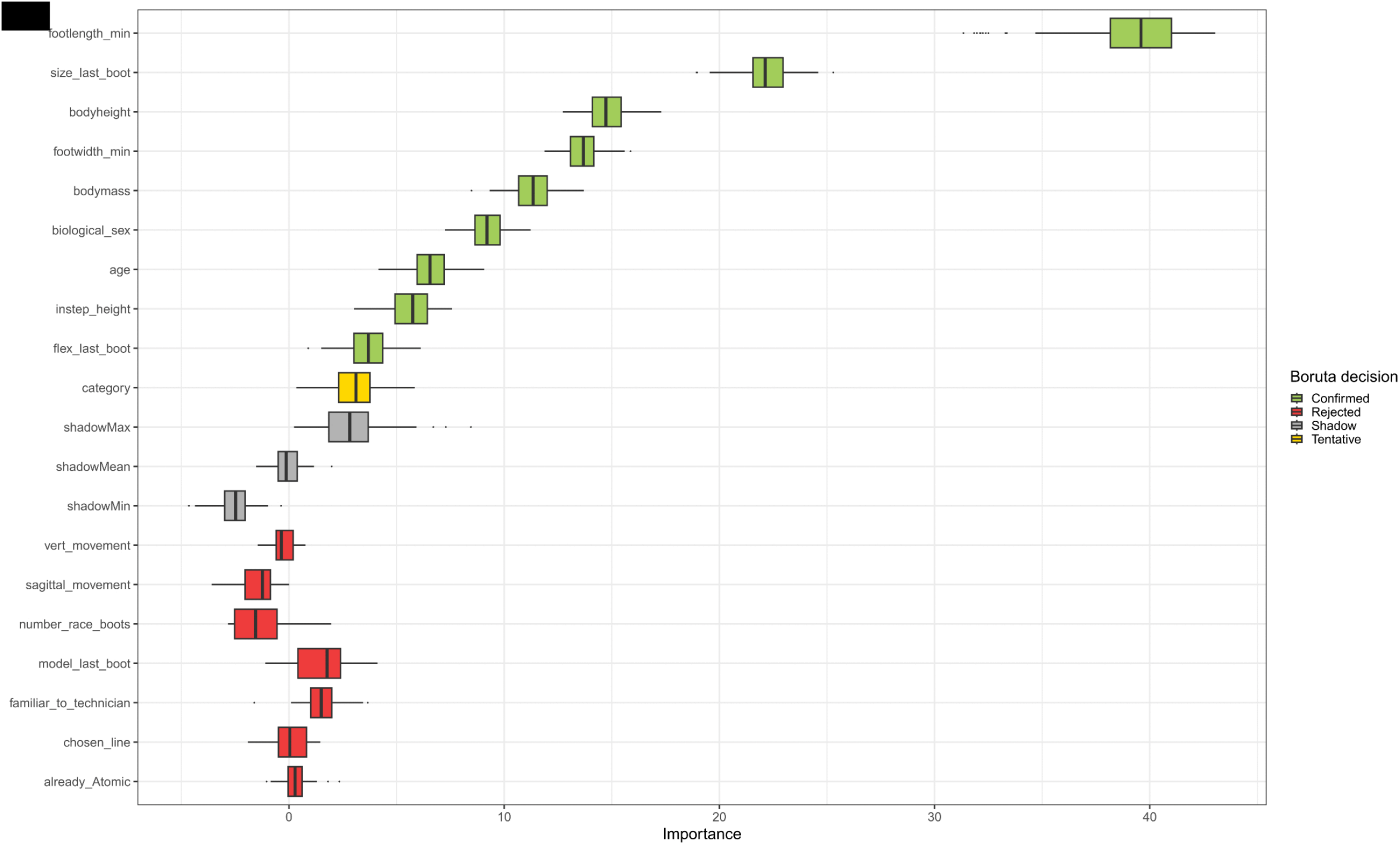
Features for the boot size. Relevant features for the boot size recommendation prediction model. The green and yellow marked variables were included in the model. The grey variables display the shadow variables of the Boruta algorithm, the red variables are not important variables.

For the boot size recommendation prediction model, in total 19 variables and 198 records were included. Three records (one of size 21.5 and two of size 28.5) were deleted as there was only one, respectively two records in the data set. After applying the Boruta algorithm 10 variables were left and used for boot size recommendation prediction (see Fig 2). The variable with the highest importance was foot length, followed by the size of the previous boot and bodyheight. The variables related to skiing style and the information, whether the last boot was also an Atomic boot were the least important variables.

Overall, the boot size prediction model achieved a mean accuracy over all five cross-validation folds of 77%, a NIR of 0.25 and a Cohen’s Kappa value of 0.86 (see Table 2).

**Fig 3:**
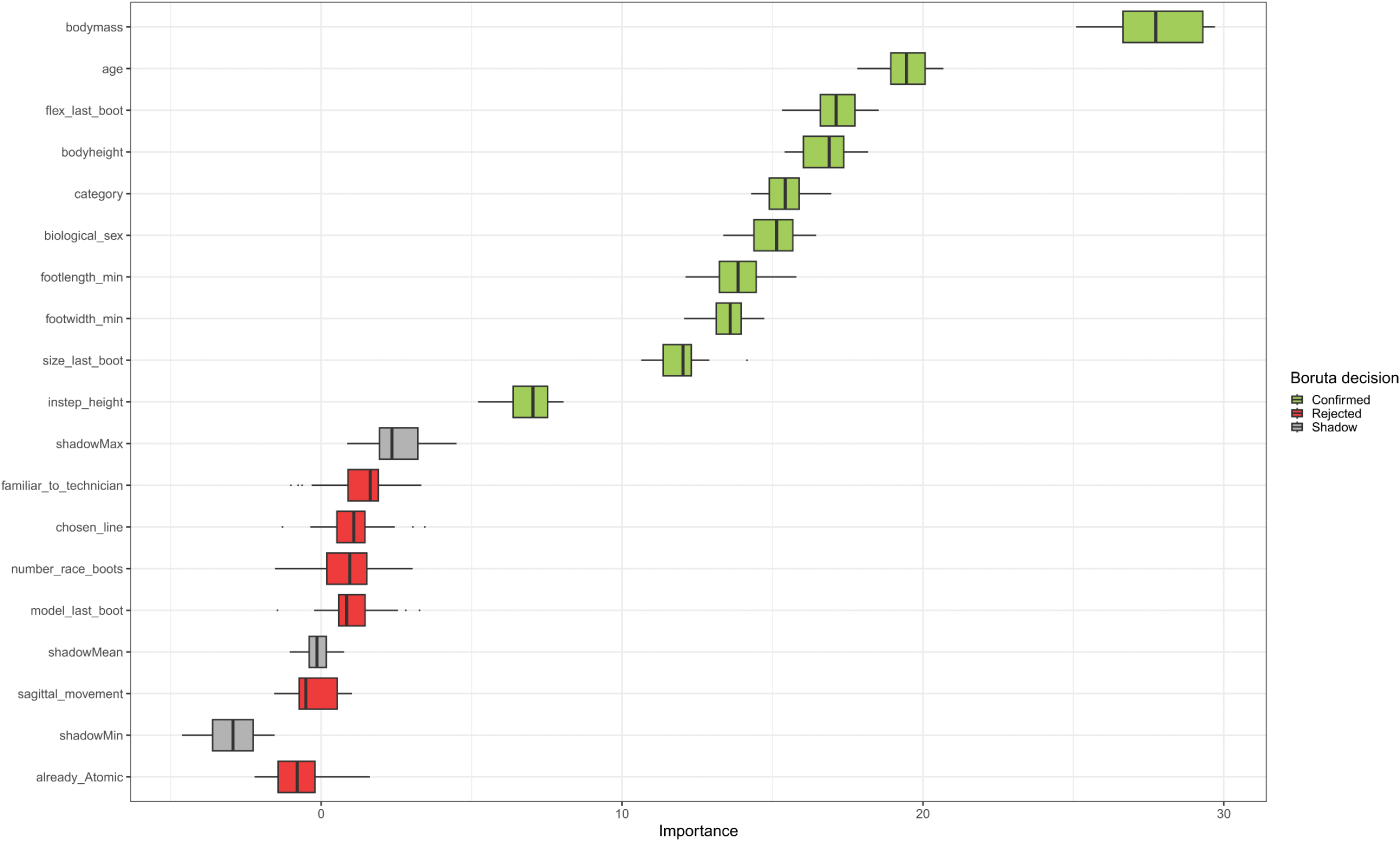
Features for the boot model. Relevant features for the boot model recommendation prediction model. The green marked variables were included in the model. The grey variables display the shadow variables of the Boruta algorithm, the red variables are not important variables.

For the boot model prediction 19 variables and 198 records were used in total. The Boruta algorithm left 10 variables in the model which are displayed in Fig 3. For the boot model prediction body mass, age and flex of the last boot were the most important variables. The variables related to skiing style and the information, whether the last boot was also an Atomic boot were also for boot model recommendation the least important variables. The boot model recommendation prediction resulted in a mean accuracy of 57%, a NIR of 0.36 and a Cohen’s Kappa value of 0.50 (see Table 2).

### Sex specific models

Prediction of boot size recommendation in female skiers required to delete two boot sizes (26.5 and 27.5) due to only one record. Boruta algorithm for the boot size recommendation in female skiers suggested five variables to be included. Foot length was the most important variable, followed by size of the previous boot, foot width, body height and body mass. For the boot model recommendation prediction, ten variables were included in the model for female skiers. The most important variable in this model was body mass, followed by age, flex of the recent boot, body height, category, foot width, foot length, instep height and size of the recent boot. Recommendation prediction models for male athletes included seven variables for boot size recommendation and nine variables for boot model recommendation. The most important variable for the boot size recommendation was foot length, followed by size of the previous boot, body height, body mass, foot width, flex of the recent boot and age. For the boot model recommendation, the most important variable was body mass, followed by category, age, body height, size of the previous boot, foot width, flex of the previous boot, foot length and instep height. The boot size recommendation model for female skiers achieved a mean overall accuracy of 75% and the boot model recommendation prediction 64%. The recommendation prediction models for male skiers achieved lower accuracy of 68% and 57% respectively (Table 2).

## Discussion

The goal of this research project was to extract experts’ knowledge, convert it into a data assessment tool and provide the expertise to others working in the field. With the underlying data of two years of data collection, it was possible to analyse boot choice recommendation criteria and find a way to base this decision on a structured, more transparent decision heuristic.

The model output should be interpreted as a prioritized suggestion. By providing probability-based rankings of the most likely boot size or model, the system is designed to support, not replace, the technician’s professional judgment. The study was based on expert recommendations, which represent the current practical standard for race-boot selection. While these recommendations include elements of subjective judgment, the aim of the modelling approach was not to eliminate expert input but to objectify and systematize it to create a more consistent and transparent decision structure. In this sense, the model formalizes the decision patterns experts apply implicitly. Translating these patterns into quantifiable variables and reproducible rules may contribute to reducing variation between technicians and improve internal consistency in decision making.

Although the overall sample size of 198 participants is modest, the use of random forest models was considered suitable for the objectives of this study. The dataset contained 19 predictor variables of mixed type, including anthropometric, equipment-related, and categorical skiing-style variables. The relationships between these predictors and expert recommendations were expected to be non-linear and potentially interacting. Random forests are well suited for such conditions, as they handle complex interactions and mixed data structures without strong parametric assumptions. Furthermore, the method provides variable importance measures that were essential for identifying key decision criteria. Nevertheless, a limited dataset increases the risk of overfitting and reduces model stability. To mitigate this risk, nested cross-validation and Boruta variable selection were applied to reduce model complexity and improve generalizability. While larger datasets would further improve robustness, the chosen approach enabled both predictive modelling and transparent feature evaluation within the available sample. Future research should therefore prioritize expanding the dataset.

The overall accuracy for the boot size prediction model revealed an accuracy of 77% with a substantial agreement and is discussed as a well applicable and feasible result.

100% of the misclassifications (for overall, male and female) were limited to ±1 size (see Supporting information). Because boot fitting in alpine skiing commonly involves fine adjustments within this range, such deviations are generally acceptable in practice. Even when the model does not predict the exact size chosen by the experts, it still guides the technician toward a realistic option. This narrow range of misclassification therefore suggests potential practical usefulness of the model.

Furthermore, the boot model prediction model showed an accuracy of 57% with a moderate agreement. These slightly worse numbers for the boot model can have numerous reasons, which should be addressed here. The concept of boot size was discussed with the experts as a way more trivial concept than the boot model. The named influencing factors by the experts for boot size recommendation were limited to foot measures (length, width, and instep height) and maturity factors like age, body height and body weight of the athletes. These assumptions prior to the data collection were well in line with the feature selection done by the Boruta algorithm (Fig 2). Additionally, the feature selection also included the variable biological sex as decisive, where detailed analysis shows, that female athletes tended to be equipped with a smaller boot compared to male athletes having the same foot length. The reason to that cannot be given by evidence, but expert feedback suggests that female athletes may prefer a tighter and therefore also smaller boot, compared to male skiers.

In contrast to the boot size prediction, the boot model choice is more complex since boot models are not as trivial distinguishable from each other as boot sizes. Influencing factors are likely based on practitioners’ and companies’ expertise, but not in terms of scientific evidence. Second, individual skiing style related parameters were discussed as important items by the experts, having an influence on skiing performance and therefore also on the boot choice. But once more, this partly intangible concept of detailed movement patterns was neither described systematically by the experts, nor scientifically in any case, in terms of the influence on human-equipment interaction. This was also found in the results of the Boruta algorithm, where items related to skiing style (chosen line, vertical movement, movement in sagittal plane) were the least important items and therefore excluded.

Overall, the low NIR values across overall and sex-specific models indicate that the observed accuracies are not driven by dominant classes but reflect meaningful predictive performance beyond a baseline model.

The discrepancy between expert ratings and Boruta feature importance for skiing-style variables suggests that complex movement patterns, such as chosen line or vertical movement, may be difficult to assess consistently across athletes. This finding highlights the need for more objective or sensor-based measures in future research as it suggests that the parameters used to operationalize skiing style in this study may not adequately capture the aspects of movement that influence boot selection. Future research should therefore aim to develop more precise and objective descriptors of skiing technique, for example using wearable sensors, pressure measurements, or video-based motion analysis.

Further, boot model characteristics cannot be compared unreflected between different companies, due to non-standardized details, like the NFI, plastique composites or shapes of the boots. There are specifications for alpine competition equipment by the FIS, but these ensure safety standards, prevent from malfunction, and do not standardize performance metrics. Therefore, specific knowledge must be developed within each company. With these reasons and influences in mind, we see the accuracy of the boot model recommendation model may still provide useful guidance. Especially with a deeper look into the miss-classified cases. For practical reasons, this makes it mandatory to provide probabilities for item recommendation, meaning as an output the boot technicians get not only one boot size or model recommendation, but two possibilities with its probabilities of agreement. Not only the experts, but also the Boruta algorithm showed the importance of biological sex aspects. To learn more about the differences between female and male athletes, sex specific models were calculated.

Although sex-specific models showed slightly higher performance for female athletes, the differences compared with the combined model were small. Given the limited dataset, maintaining a larger pooled sample increases model stability and reduces the risk of overfitting. This creates a trade-off between model specificity and robustness. In the present study, the marginal improvement of the female-specific model did not outweigh the benefits of the unified model. Future studies with larger datasets may enable more reliable sex-specific modelling.

### Limitations and outlook

Even though the presented process of equipment choice in alpine ski racing as a precondition to human-equipment interaction is described for the first time and can be adapted to various use cases (e.g., other brands, other type of equipment, other sports), the presented results apply only for the small cohort of ski racers of one brand.

The datasheet used in this study was developed through a structured Delphi-inspired process involving experienced boot technicians. This ensured expert consensus on the relevance of included items. However, the instrument was not designed or validated as a psychometric questionnaire. Its purpose was to operationalize expert decision criteria into a standardized data collection tool. Currently, no published instruments exist that systematically capture parameters relevant for alpine ski boot selection, meaning no validated reference questionnaire was available. This highlights the exploratory nature of the present work. Future research should evaluate reliability, for example through inter-rater agreement among technicians, and assess content validity using larger expert panels or emerging research on human–equipment interaction in alpine skiing.

The reduction of the original 5-point Likert scale to three levels for skiing-style variables reflects the simplified decision structure used in practice. However, this reduction decreases granularity and may mask subtle differences. Future studies with larger datasets could retain the full five-level scale or apply ordinal modelling approaches.

Although nested cross-validation represents a rigorous internal validation strategy for datasets of this size, it only estimates model performance within the same underlying sample. The limited number of cases did not allow the creation of an independent test set without reducing model stability. Consequently, external generalizability cannot be claimed. Future studies should validate the models using temporally or geographically independent datasets.

Another limitation relates to the data source. All observations were collected at one company’s performance center. As a result, the model reflects the fitting practices, product characteristics, and athlete population of this manufacturer. Elite athletes, who often use highly individualized or prototype equipment, may follow different decision processes. Furthermore, boot characteristics such as NFI, plastic composition, and geometry are not standardized across manufacturers. Since each company defines stiffness ratings internally, boots with identical NFI labels may differ substantially between brands. This limits the direct transferability of the derived model to other manufacturers. While the model itself should not be assumed to apply across different equipment ecosystems without further validation, the methodological framework is transferable. The structured extraction of expert knowledge, translation into measurable parameters, and subsequent development of predictive models could be adapted to other brands, where boot geometries, stiffness measures, or material properties differ. Applying this approach to a wider range of athletes, including elite skiers, recreational skiers, or youth development programs, may help standardize and objectify equipment selection processes in diverse contexts. Expanding the framework to these broader populations could also help identify whether decision criteria are brand-specific or whether common patterns emerge across different equipment ecosystems.

Potential confounding factors may also have influenced the expert recommendations used as ground truth. For example, practical constraints such as available boot inventory at the time of fitting or financial considerations of the athletes were not captured in the dataset. Further, class imbalance remains a limitation, as uneven class distributions can bias predictions toward more frequent categories. Although rare classes were removed and Cohen’s Kappa was used to account for chance agreement, residual imbalance may still affect model performance.

Additionally, we cannot prove by evidence yet, whether the recommended boot is also the best performing boot, neither in terms of safety, nor in terms of performance optimization, as the model predicts expert-derived choices rather than objectively verified performance.

Future studies should incorporate on-snow validation by comparing model-based recommendations with expert-selected boots in terms of skiing performance, biomechanical measures, and potential injury-related outcomes. Such validation is necessary to establish whether objective recommendation systems provide tangible benefits in real skiing environments.

## Conclusion

The goal of this research project was, to add knowledge to the field of human-equipment interaction in alpine ski racing and to gain knowledge about the criteria how to choose an alpine skiing racing boot. The proposed model translates expert-derived boot selection decisions into a structured and reproducible framework using measurable input variables. Rather than replacing expert judgment, the model reproduces expert decision patterns in a transparent and systematic way and provides probability-based recommendations to support technicians in the boot fitting process.

With the database of 198 cases, boot recommendation models for boot size and boot model were calculated. The accuracy and Cohen’s Kappa value were better for the boot size than the boot model without any sex differences. This might be due to the more complex concept of boot model than boot size. Additionally, analysis of wrongly predicted cases revealed to be miss-classified by only one level (e.g., one size bigger or smaller than the actual boot). This limited deviation aligns with practical boot-fitting tolerances and underscores that the model offers actionable support for narrowing down appropriate boot sizes.

With this small range in mind, it was possible to build an acceptable model for boot recommendations in alpine ski racing, which can help boot technicians of this specific brand in boot choice selection.

## Supporting information

S1 Data Collection sheet. (PDF)

S2 Hyperparameters. (PDF)

S3 Misclassifications. (PDF)
