# Supplementary material for: From expert opinion to data driven selection of sports equipment: Boot selection in alpine ski racers
Source: PLoS One. 2026 Jun 4;21(6):e0349862. doi: 10.1371/journal.pone.0349862 (PMC13235891; doi:10.1371/journal.pone.0349862)
Supplement: S1 Data — (PDF) [file pone.0349862.s001.pdf]

APPENDIX

Part 1 – Data collection sheet

Boot Choice

Date: ____________________________

Place: ____________________________

Technician: ____________________________

(1)Sex	**□ male**			**□ female**		
(1)Code						
(1)Date of birth						
(2)Bodymass (kg)			(2)Bodyheight (cm)			
	(1)**How many** Atomic race boots fitted by a boot technician did you have up to now? **□ 0** **□ 1-3** **□ >3**					
(3)Recent boot	**Model**		**Flex**		**Size**	
(4)Footlength (mm)	**Left:**			**Right:**		
(4)Footwidth (mm)	**Left :**			**Right :**		
(4)Instep height (mm)	**Left :**			**Right :**		
(1)Category	**□ WC/ EC**<br>**□ Masters- race-oriented**<br>**□ U14 / U16**			**□ Youth / FIS**<br>**□ Masters – piste-oriented**<br>**□ U10 / U12**		
(1)Main discipline / purpose	**□ Technical disciplines**			**□ Speed disciplines**		**□ Piste**
(5)How well do you as a boot technician know the athlete?						
**□ very good** **□ ordinary** **□ not at all**						
(6)In comparison to **all athletes in your agegroup** your chosen **line** can be described as:						
**□ very direct** **□ rather direct** **□ neutral** **□ rather round** **□ very round**						
(6)In comparison to **all athletes in your agegroup** your body position **forwards and backwards** can be described as:						
**□ far backwards** **□ rather backwards** **□ neutral** **□ rather forwards** **□ far forwards**						
(6)In comparison to **all athletes in your agegroup**, your **vertical movement** (up-down or down-up) can be described as:						
**□ very pronounced** **□ rather pronounced** **□ neutral** **□ rather weak** **□ very weak**						

(7)Recommended boot			**Size**							
□TI 170	□TI 150	□TI 130	□TI 110	□ STI 150	□ STI 130	□ STI 110	□ STI LC 110	□ STI LC 90	□ STI LC 70	□other

(8)Condition / Adaptation (optional)

Further notes and comments

PARIS LODRON UNIVERSITÄT SALZBURG

Sport- und Bewegungswissenschaft/USI

Digital Motion
in Sports, Fitness & Well-being
