# Supplementary material for: From expert opinion to data driven selection of sports equipment: Boot selection in alpine ski racers
Source: PLoS One. 2026 Jun 4;21(6):e0349862. doi: 10.1371/journal.pone.0349862 (PMC13235891; doi:10.1371/journal.pone.0349862)
Supplement: S2 — (PDF) [file pone.0349862.s002.pdf]

Hyperparameters

S2 Hyperparameters. Table 1 displays the mtry value for each random forest model. The number of trees (ntree) was always set to 500.

Table 1. Hyperparameters of all recommendation prediction models

	**Overall**		**Female**		**Male**	
**mtry**	**Size**	**Model**	**Size**	**Model**	**Size**	**Model**
Fold 1	20	2	2	11	8	2
Fold 2	11	20	5	11	8	11
Fold 3	11	11	3	21	8	11
Fold 4	11	2	5	2	8	21
Fold 5	11	2	2	12	15	1
