# Supplementary material for: From expert opinion to data driven selection of sports equipment: Boot selection in alpine ski racers
Source: PLoS One. 2026 Jun 4;21(6):e0349862. doi: 10.1371/journal.pone.0349862 (PMC13235891; doi:10.1371/journal.pone.0349862)
Supplement: S3 — (PDF) [file pone.0349862.s003.pdf]

Misclassifications

S3 Table. Misclassifications.

Table 1. Misclassifications — Boot size recommendation prediction model (overall)

Fold 1		Fold 2		Fold 3		Fold 4		Fold 5	
**Pred.**	**True**	**Pred.**	**True**	**Pred.**	**True**	**Pred.**	**True**	**Pred.**	**True**
23.5	24.5	24.5	25.5	25.5	26.5	25.5	26.5	23.5	24.5
22.5	23.5	25.5	24.5	27.5	26.5	24.5	25.5	24.5	23.5
23.5	24.5	23.5	22.5	26.5	27.5	24.5	25.5	26.5	27.5
23.5	24.5	23.5	24.5	24.5	23.5	24.5	23.5	23.5	24.5
25.5	24.5	25.5	26.5	25.5	26.5	22.5	23.5	25.5	26.5
24.5	25.5	23.5	24.5	22.5	23.5	23.5	22.5	26.5	25.5
25.5	24.5	26.5	25.5	24.5	25.5	23.5	24.5	27.5	26.5
		26.5	25.5	25.5	26.5			23.5	22.5
		24.5	23.5	27.5	26.5			23.5	24.5
		24.5	23.5	25.5	24.5			26.5	25.5
				24.5	23.5			24.5	25.5

Table 2. Misclassifications — Boot size recommendation prediction model (females)

Fold 1		Fold 2		Fold 3		Fold 4		Fold 5	
**Pred.**	**True**	**Pred.**	**True**	**Pred.**	**True**	**Pred.**	**True**	**Pred.**	**True**
24.5	25.5	23.5	24.5	23.5	24.5	22.5	23.5	24.5	23.5
22.5	23.5	24.5	25.5	24.5	25.5	23.5	22.5	23.5	22.5
24.5	25.5	23.5	22.5			22.5	23.5	24.5	25.5
25.5	24.5					23.5	24.5	25.5	24.5
								25.5	24.5
								23.5	22.5

Table 3. Misclassifications — Boot size recommendation prediction model (males)

Fold 1		Fold 2		Fold 3		Fold 4		Fold 5	
**Pred.**	**True**	**Pred.**	**True**	**Pred.**	**True**	**Pred.**	**True**	**Pred.**	**True**
27.5	26.5	27.5	26.5	25.5	26.5	25.5	26.5	24.5	23.5
24.5	25.5	26.5	25.5	25.5	26.5	26.5	27.5	23.5	24.5
26.5	25.5	26.5	25.5	25.5	24.5	24.5	25.5	25.5	26.5
27.5	26.5	25.5	24.5	24.5	25.5	23.5	24.5	26.5	27.5
24.5	23.5	23.5	24.5	23.5	24.5	24.5	23.5	23.5	24.5
22.5	23.5	24.5	23.5	24.5	23.5	25.5	26.5	26.5	25.5
25.5	26.5	26.5	25.5			26.5	27.5	26.5	27.5
27.5	26.5					25.5	26.5		
						24.5	25.5		
						25.5	26.5		

Table 4. Misclassifications — Boot model recommendation prediction model

**Fold 1**		**Fold 2**		**Fold 3**		**Fold 4**		**Fold 5**	
**Pred.**	**True**	**Pred.**	**True**	**Pred.**	**True**	**Pred.**	**True**	**Pred.**	**True**
STI 130	STI 150	TI 170	TI 150	STI LC 90	STI 110	TI 110	STI 130	TI 150	TI 170
STI LC 90	STI 110	STI 130	STI 150	STI LC 90	STI LC 110	TI 150	TI 170	STI 130	STI LC 110
TI 150	TI 130	STI 130	STI LC 110	TI 130	TI 110	STI 130	STI 130	STI 130	STI 150
TI 170	TI 150	STI LC 90	STI LC 110	STI LC 70	STI LC 90	STI LC 90	STI LC 70	STI 110	STI LC 110
STI LC 90	STI LC 70	STI 110	TI 130	STI LC 70	STI LC 90	STI LC 90	STI LC 110	TI 130	TI 150
STI LC 90	STI LC 110	STI LC 70	STI LC 90	STI LC 90	STI LC 70	STI LC 90	STI LC 70	STI 150	STI 130
TI 130	STI 130	STI 110	STI LC 90	STI LC 70	STI LC 90	STI 130	STI LC 110	STI 130	STI 130
STI LC 70	STI LC 90	STI LC 70	STI LC 90	STI LC 90	STI LC 70	TI 170	TI 150	TI 110	STI 110
TI 150	STI 150	STI 130	STI 110	STI LC 90	STI LC 70	STI LC 90	STI 110	STI 110	STI LC 90
STI 130	STI 150	TI 110	STI 130	TI 170	TI 150	STI 130	STI 150	STI 130	STI 150
STI 110	STI 110	STI LC 90	STI LC 70	STI 130	TI 110	STI LC 90	TI 110	TI 130	TI 150
STI 130	STI 110	TI 150	TI 170	TI 170	TI 150	TI 110	STI 110	TI 110	STI 110
STI 130	STI 150	TI 110	STI 130	STI 130	TI 130	STI 150	STI 130	STI 110	STI 110
TI 130	TI 150	TI 150	TI 170	STI 130	STI 150	STI LC 90	STI LC 70	STI LC 70	STI LC 90
		STI LC 90	STI 110	STI LC 70	STI LC 90	STI LC 70	STI LC 90	TI 150	TI 170
		TI 110	STI 130	TI 110	STI 150	STI 150	STI 130		
		TI 150	TI 170	STI LC 90	STI 110	STI LC 90	STI LC 70		
		STI LC 70	STI LC 90			STI LC 70	STI LC 90		
		STI 110	STI 130						

Table 5. Misclassifications — Boot model recommendation prediction model (females)

**Fold 1**		**Fold 2**		**Fold 3**		**Fold 4**		**Fold 5**	
**Pred.**	**True**	**Pred.**	**True**	**Pred.**	**True**	**Pred.**	**True**	**Pred.**	**True**
STI LC 70	STI LC 90	STI 130	STI LC 110	STI LC 90	STI LC 110	STI 130	STI 150	STI LC 110	STI 110
STI LC 90	STI LC 70	STI 150	STI 130	STI LC 110	STI LC 90	STI 130	STI 150	STI LC 70	STI LC 90
STI 130	STI 110	STI LC 70	STI LC 90	STI 150	STI 130	STI 110	STI LC 110	STI LC 90	STI LC 70
STI 150	STI 130	STI LC 70	STI LC 90	STI 130	STI 110	STI 130	STI 150	STI 130	STI 150
		STI LC 70	STI LC 90	STI LC 70	STI LC 90	STI LC 110	STI 130	STI 130	STI 150
		STI 150	STI 130			STI 130	STI 110	STI LC 70	STI LC 90
		STI 130	STI 150						

Table 6. Misclassifications — Boot model recommendation prediction model (males)

**Fold 1**		**Fold 2**		**Fold 3**		**Fold 4**		**Fold 5**	
**Pred.**	**True**	**Pred.**	**True**	**Pred.**	**True**	**Pred.**	**True**	**Pred.**	**True**
TI 150	TI 170	STI LC 70	STI LC 90	TI 130	TI 110	TI 150	TI 170	TI 110	STI 130
TI 110	STI 110	STI LC 90	STI LC 70	STI 130	TI 130	STI LC 70	STI LC 90	TI 150	TI 130
TI 110	STI 130	STI LC 90	STI LC 110	STI LC 90	STI LC 110	TI 150	TI 130	TI 170	TI 150
STI 130	TI 110	TI 170	TI 150	STI LC 90	STI LC 70	STI LC 90	STI LC 110	STI 110	STI LC 90
STI 130	STI 150	TI 110	STI 130	TI 130	TI 150	TI 170	TI 150	TI 170	TI 150
STI 150	TI 170	TI 150	STI 150			TI 110	TI 130	STI LC 90	STI 110
TI 110	STI 110	STI LC 90	STI 110			STI LC 70	STI LC 90	TI 130	STI 150
TI 110	STI 110	STI LC 70	STI LC 90			TI 170	TI 150	STI LC 90	STI LC 70
		TI 150	TI 170			STI LC 70	STI LC 90	TI 150	TI 170
		STI 110	STI 110			TI 110	STI 110	TI 110	STI 110
		TI 150	TI 170			STI 110	TI 110		
		STI LC 70	STI LC 90			TI 110	STI 130		
		STI 150	TI 110			STI 110	TI 110		
						TI 130	STI 150		
						TI 110	STI 150		
